# Laponites^®^ for the Recovery of ^133^Cs, ^59^Co, and ^88^Sr from Aqueous Solutions and Subsequent Storage: Impact of Grafted Silane Loads

**DOI:** 10.3390/ma13030572

**Published:** 2020-01-25

**Authors:** Thomas Thiebault, Jocelyne Brendlé, Grégoire Augé, Lionel Limousy

**Affiliations:** 1 IS2M, Université de Haute-Alsace, CNRS, UMR 7361, 3b rue Alfred Werner, F-68100 Mulhouse, France; jocelyne.brendle@uha.fr (J.B.); lionel.limousy@uha.fr (L.L.); 2 Université de Strasbourg, F-67081 Strasbourg, France; 3 EPHE, PSL University, UMR 7619 METIS (SU, CNRS, EPHE), 4 Place Jussieu, F-75005 Paris, France; 4 ONET Technologies, 36 Boulevard de l’Océan, CS 20280, 13258 Marseille CEdEX 09, France; gauge@onet.fr

**Keywords:** adsorption, silane, radionuclides, LAPONITE®, desorption

## Abstract

In this study, silylated Laponites^®^ (LAP) were synthetized with various loads of 3-aminopropyltriethoxysilane (APTES) to evaluate their adsorption properties of ^133^Cs, ^59^Co, and ^88^Sr during single-solute and competitive experiments. The increase in the initial load of APTES increased the adsorbed amount of APTES in the resulted grafted clay. The characterization of LAP-APTES exhibited a covalent binding between APTES and LAP and emphasized the adsorption sites of APTES for each tested load. In comparison with raw LAP, LAP-APTES displayed significantly higher adsorption properties of Co^2+^, Cs^+^, and Sr^2+^. The competitive adsorption of these three contaminants provides a deeper understanding of the affinity between adsorbate and adsorbent. Therefore, Co^2+^ displayed a strong and specific adsorption onto LAP-APTES. Except for Cs^+^, the adsorption capacity was improved with increasing the load of APTES. Finally, the desorption behavior of the three contaminants was tested in saline solutions. Cs^+^ and Sr^2+^ were significantly released especially by inorganic cations displaying the same valence. Conversely, desorption of Co^2+^ was very low whatever the saline solution. LAP-APTES, therefore, presented suitable adsorption properties for the removal of radionuclides especially for Co^2+^, making this material suitable to improve the decontamination of radioactive wastewaters.

## 1. Introduction

Low-level radioactive wastes (LLRW) are mostly generated by the operation of nuclear power plants [1]. LLRW contain various radionuclides and especially fission by-products. Among them, ^60^Co, ^137^Cs, and ^90^Sr are considered as particularly hazardous due to their important solubility and long half-lives [2,3]. The decontamination of LLRW represents an important challenge due to the hardness to treat such complex effluents with economically viable solutions. Reaching a suitable process for the decontamination of LLRW is therefore of high concern in order to avoid the environmental dispersion of hazardous radioactivity [4,5]. In this aim, adsorption could represent an easy way to adsorb radioactive inorganic cations if the selected adsorbent presents both appropriate adsorption properties and reasonable costs. Several materials and processes were already tested in order to adsorb radionuclides, such as zeolites, oxides, membranes, and clay-based adsorbents [6,7,8,9,10,11,12].

Natural clay minerals are abundant and cheap materials renowned for their adsorption capacity of a wide range of contaminants, especially due to their cation exchange capacity (CEC) [13,14,15,16]. However, this adsorption capacity and/or selectivity is limited for the removal of several contaminants such as non-cationic contaminants [17,18,19]. To improve the adsorption properties of clay minerals, their organo-modification with various organic compounds, such as surfactants or organoalkoxysilane, was extensively studied in recent years [20,21,22,23,24,25]. One of the limitations for the use of surfactants is the possible substitution of the compensating inorganic cations in the interlayer space by positively charged surfactants moieties through cation exchange. As a result, this important property of clay minerals can be hindered even if the hydrophobicity of clay minerals was increased [26,27]. The resulted organo-clays usually displayed better adsorption capacities of anionic and neutral species, although cationic species are only poorly adsorbed [17,28]. However, to maximize the potential of clay-based adsorbents, it appears useful to maintain their cation exchange capacity. As a result, the silane grafting reaction (i.e., silylation) on clay minerals allows a stable immobilization of organic moieties by covalent binding with clay mineral with a weak impact on its interlayer space [29,30,31]. This chemical reaction therefore preserves the CEC and prevents from the potential secondary pollution generated by the weak bonding between some toxic surfactants and clay minerals [32,33]. Depending on the grafting agent, the silylation process can introduce specific chemical functions that could strongly improve the adsorption selectivity for the targeted contaminants [34,35,36,37]. Among the possible silylating agents, 3-aminopropyltriethoxysilane (APTES) has already demonstrate its great versatility for the adsorption of various contaminants [30,38,39,40].

The main objective of this work is therefore to investigate the adsorption of stable isotopes (i.e., ^133^Cs, ^59^Co, and ^88^Sr) of three commonly detected radionuclides in LLRW, ^137^Cs, ^90^Sr, and ^60^Co, onto a synthetic hectorite (LAPONITE^®^ RD, LAP), which was used raw or grafted with APTES. The impact of grafting load on the adsorption properties of Co^2+^, Sr^2+^, and Cs^+^ of silylated clay minerals (LAP-APTES) was investigated as well as the competitive effect for adsorption and the desorption behavior of contaminants in various type of saline solutions.

## 2. Materials and Methods

### 2.1. Chemical Reagents

LAPONITE® RD (LAP) (purchased to BYK Additives & Instruments, Moosburg, Germany) was used without further modification. LAP is a synthetic hectorite with almost octahedral substitutions with the following structural formula Na^+^_0.7_[(Si_8_Mg_5.5_Li_0.3_)O_20_(OH)_4_]^−0.7^. Grafting agent, 3-aminopropyltriethoxysilane was supplied by Sigma-Aldrich Company (Steinheim, Germany) assuming a purity grade up to 99%. General information about APTES is presented in Table S1.

Cesium chloride (CsCl, >99.99%), strontium hexahydrated chloride (SrCl_2_·6H_2_O, >99%), and cobalt hexahydrated chloride (CoCl_2_·6H_2_O, >98%) were supplied by Carl Roth Company (Karlsruhe, Germany).

Chemical reagents such as HCl, NaOH, absolute ethanol, NaCl and CaCl_2_ were supplied by Sigma-Aldrich Company (Steinheim, Germany) at analytical grade and were used without further modifications.

### 2.2. Synthesis of Grafted-LAP

The protocol for the grafting of LAP can be detailed as follows. LAP (6 g) was mixed with various amounts of APTES relating to the cation exchange capacity (CEC) of LAP (i.e., 75 meq. 100 g^−1^) in absolute ethanol (200 mL). Five different APTES loads were used corresponding to 1, 2, 3, 4, and 10 CEC of LAP, respectively. After the addition of APTES, the suspension was continuously stirred with magnetic stirrer at 200 rpm during 24 h, at 60 °C under Ar atmosphere.

The material was then recovered by centrifugation at 8000 rpm during 10 min. The material was rinsed two times with absolute ethanol and then two times with pure water and recovered between each rinse step by centrifugation at 8000 rpm during 10 min. Then, the material was dried in an oven at 60 °C for 72 h.

LAP-APTES are hereafter labeled according to the APTES load used during synthesis.

### 2.3. Experimental Techniques

Fourier Transformed Infrared (FTIR) spectroscopy measurements in the range 400 to 4000 cm^−1^, were recorded using a Bruker 55 FT-IR (Bruker Company, Fällanden, Switzerland) spectrometer equipped with Nd:YAG laser operating at 1064 nm and a Ge detector. The analyses were performed in transmission mode and each spectrum was the average of 256 scans collected at 2 cm^−1^ resolution.

X-Ray Diffraction (XRD) patterns of the different samples were recorded on a PANalytical X’Pert Pro MPD diffractometer (PANanalytical Company, Almelo, Netherlands) using Cu Kα radiation (λ = 1.5418 Å) and Θ-2Θ mounting (Bragg-Brentano geometry). Measurements were achieved for 2Θ angles values between 2° and 70°, step 0.17° 2Θ. 

Thermogravimetric analyses (TG) were performed on a TG Mettler Toledo STARe apparatus (Mettler Toledo Company, Greifensee, Switzerland) under air flow with a heating rate of 2 °C min^−1^ from 30 to 900 °C. All samples were air-dried during 48 h in order to obtain comparable hydration state.

Zeta potential (ZP) measurements were realized using a Malvern Nanosizer instrument (Malvern Company, Malvern, UK). Twenty milligrams of material was dispersed in 50 mL of a 10^−3^ M NaCl solutions. The pH of the suspension was adjusted using 0.1 M NaOH and/or HCl solutions and the suspension was stirred for 24 h until ionic equilibrium was reached.

The concentrations of the different cations (Cs^+^, Co^2+^, and Sr^2+^) were quantified with atomic absorption spectroscopy (Varian AA 240 FS, Varian Company, Palo Alto, CA, USA), using the absorption mode and acetylene as fuel.

Solid-state ^1^H (I = 1/2) magic angle spinning (MAS) Nuclear MagneticR (NMR) experiments were performed at room temperature on a Bruker Avance II 400 spectrometer (Bruker Company, Fällanden, Switzerland) operating at B0 = 9.4 T (Larmor frequency ν0 = 400.17 MHz). Single pulse experiment was recorded with a double channel 2.5 mm Bruker MAS probe, a spinning frequency of 30 kHz, a π/2 flip angle, pulse duration of 3.5 μs and a recycling delay of 35 s.

^1^H decoupled ^29^Si MAS and ^1^H- ^29^Si cross-polarization (CP)MAS NMR spectra were recorded on a Bruker Avance II 400 spectrometer (Bruker Company, Fällanden, Switzerland) (B0 = 9.4 T) operating at 79.460 and of 100.484 MHz, respectively. Samples were packed in a 7 mm diameter cylindrical zirconia rotor and spun at a spinning frequency of 4 kHz. A π/6 flip angle, pulse duration of 6.5 μs, recycle delay of 80 s was used for ^1^H decoupled ^29^Si MAS NMR, whereas a π/2 flip angle, pulse duration of 6.5 μs, contact time of 4 ms, and a recycle delay of 10 s were used for CP MAS.

Decompositions of the NMR spectra to extract the proportion of the corresponding species were performed with the DMfit software [41].

### 2.4. Adsorption Experiments

Adsorption experiments were carried out both in single-solute and competitive solutions. Typically, the adsorbent mass was 100 mg in 100 mL of solution spiked with various concentrations of contaminant. The concentration of each contaminant ranged from 6 to 600 µM for Cs^+^, 4.2 to 420 µM for Co^2+^ and 3.7 to 370 µM for Sr^2+^ in both single-solute and competitive solutions (7 concentrations). Two additional concentrations for the single-solute adsorption of Co^2+^ were added in order to reach the saturation of the adsorbent, i.e., 1.05 and 2.10 mM, respectively. The final suspension was spiked at 0.1 mM of NaCl and let in interaction during 24 h at room temperature (293 K). This duration was previously determined based on kinetic experiments (data not shown), in which the adsorption was completed after 5 min of contact. The pH value of each suspension was not adjusted, but controlled at the beginning and at the end of interaction period of 24 h (i.e., pH values ranged between 6 and 6.5). Suspensions were then centrifuged at 8000 rpm during 5 min and the contaminant concentration was determined by AAS in supernatant.

The impact of pH on the adsorption capacity of Cs^+^, Co^2+^ and Sr^2+^ in competition (50 ppm of each salt) was characterized with the same protocol on LAP and LAP-APTES-4CEC. The pH of the suspension was modified with 1 M HCl and 1 M NaOH solutions to obtain a range of pH values between 2 and 12, two times adjusted to avoid the buffering effect of LAP.

### 2.5. Desorption Experiments

After interaction with the highest starting concentration of contaminants in competitive experiment, adsorbents were recovered by centrifugation at 8000 rpm during 10 min. The recovered material was then dried at 60 °C during 72 h. Twenty milligrams of dried adsorbent was then mixed with 20 mL of three different saline solutions: pure water, 1 mM of CaCl_2_, and 2 mM of NaCl. The suspension was then gently stirred during one week at room temperature. Suspensions were then centrifuged at 8000 rpm during 10 min and the contaminant concentration was determined by AAS in supernatant.

### 2.6. Sorption Modeling

The fit of the resulting adsorption isotherms by using Langmuir, Freundlich, and Dubinin–Radushkevich (DR) equation models drives to numerous thermodynamic parameters allowing one to precisely quantify the affinity of contaminants with the sorbent. Briefly, Langmuir model is expressed by the following equation,

$$q_{e}=\frac{q_{max}K_{L}C_{e}}{1+K_{L}C_{e}}$$

where $q_{e}$ is the equilibrium amount adsorbed on sorbent (mmol g^−1^), *C_e_* the equilibrium concentration in the supernatant (mmol L^−1^), *q_max_* the adsorption capacity of the sorbents (mmol g^−1^), and *K_L_* is the Langmuir adsorption constant (L mmol^−1^), which is related to the free energy (ΔG°) of adsorption. The linear Freundlich model equation is written as

$$lnq_{e}=lnK_{F}+\frac{1}{n}lnC_{e}$$

where *K_F_* (L g^−1^) and *n* are the Freundlich constants indicating the extent of the adsorption and the degree of nonlinearity between contaminants and the adsorbent respectively. The DR equation can be expressed in its linear form as

$$lnq_{e}=lnq_{max}+\beta \varepsilon^{2}$$

where ε corresponds to the Polanyi potential whose relation including *C_e_* (the equilibrium concentration) could be found elsewhere. The constant β corresponds to the activity coefficient associated to the mean free energy *E* (kJ mol^−1^) by the equation:

$$E={\left(2\beta \right)}^{-1/2}$$

This parameter gives information whether the adsorption mechanism involves a chemisorption or physical adsorption. Indeed, if the magnitude of *E* is below 8 kJ mol^−1^, physisorption is envisaged, while for *E* > 8 kJ mol^−1^ the adsorption process follows chemisorption.

## 3. Results and Discussion

### 3.1. Characterization of LAP-APTES

The FTIR spectrum of LAP exhibits various bands that can be assigned to Si–O, Si–O–Si, and Mg_3_–OH at 1002, 693, and 662 cm^−1^, respectively (Figure S1), which are representative of the structural organization of LAP [42]. The spectrum of LAP also displays a broad band due to the presence of physisorbed water at ~3450 cm^−1^, and a small shoulder at 3680 cm^−1^, which can be assigned to surface hydroxyls groups (Figure S1). FTIR spectra of LAP-APTES show typical features characteristics of the organic compounds: the absorption bands at 2880–2940 cm^−1^ relative to the symmetric and antisymmetric CH_2_ stretching vibrations of APTES as well as the absorption bands between 1350 and 1600 cm^−1^ which are related to CO_2_/CH_2_ vibrations [30,43]. These specific bands testify the proper grafting of APTES onto LAP. Even if these analyses are only qualitative, the relative increase of absorption band intensity with increasing the load of APTES can be noticed (Figure S1).

**Figure 1:**
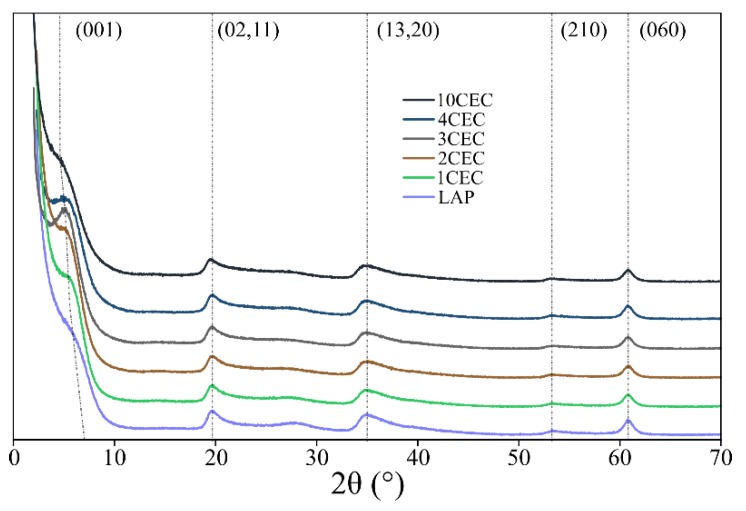
X-ray diffraction patterns of LAP and LAP-APTES for different loads of grafting agents.

XRD patterns of LAP and LAP-APTES are presented in Figure 1. The XRD pattern of LAP shows a series of reflections at 7.0, 19.4, 34.2, and 61° 2Θ degrees, which are attributed to (001), (02,11), (13,20), and (060) reflections of the synthetic products, respectively. The only variation between LAP and LAP-APTES is the shift to lower angular values of the reflection associated to d_001_ plan with increasing the load of APTES. The calculated basal spacings are 13.6, 14.9, 15.6, 16.1, 16.3, and 16.6 Å for LAP and LAP-APTES loaded with 1, 2, 3, 4, and 10 CEC of grafting agents respectively (Figure 1). This slight increase of the basal spacing could be related to the conformation of grafting agents (i.e., the formation of polysiloxane multi-layers), or to the possible penetration of the interlayer space by APTES [44,45].

TG analyses allow assessing the loss of weight of the adsorbent during a gradual heating. Generally, organo-modified clay minerals display three main weight losses during heating. The first one is associated to the evaporation of free and adsorbed water, ranging between the initial temperature and 150 °C. The second one is related to the thermal oxidation of intercalated/grafted organic compounds between 150 and 600 °C with a maximal decomposition temperature depending on the characteristic of organic moieties. Finally, for temperature higher than 600 °C, only the dehydroxylation of clay minerals is expected [46].

**Figure 2:**
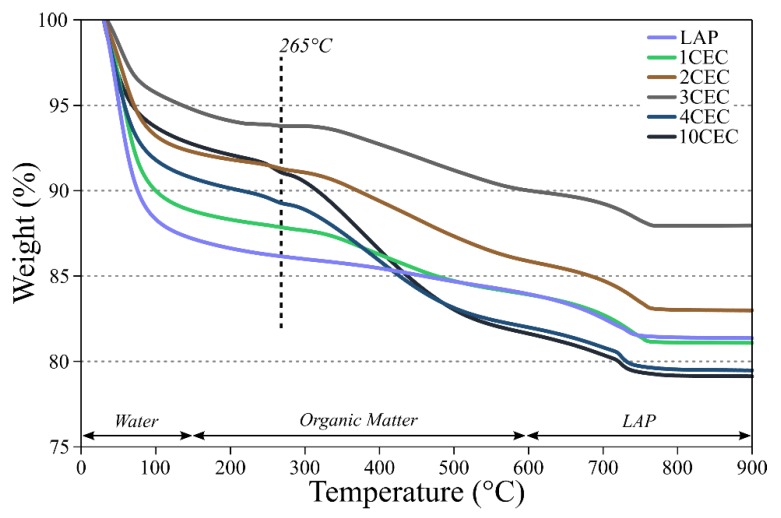
TG curves of LAP and LAP-APTES between 30 and 900 °C with a heating ramp of 2 °C·min^−1^.

TG analyses of both LAP and LAP-APTES were conducted by two distinct experimental sequences. The water content will therefore not be further discussed due to the variation in the surrounding moisture between the two sequences, affecting the derived water content in the domain of the thermal oxidation of organic matter (i.e., 150–600 °C) two main weight losses are displayed (Figure 2). The first one at 265 °C, only visible at 3 CEC and higher loads of APTES, and the second one at ~380 °C (Figure S2). These two distinct losses have been discussed in the literature and especially the former. Several assumptions assigned this weight loss to the decomposition of physisorbed APTES in the interlayer space [47], or to the initial combustion of aminopropyl moieties [31,43]. From our results, it is not possible to deeper understand these assumptions.

However, this loss of weight is clearly associated with the presence of APTES onto clay minerals and not with intercalated moieties as in other clay minerals [45,46]. On the other hand, the weight loss around 380 °C is attributed to the thermal oxidation of covalently bonded APTES onto LAP [37,48]. DTG curves are available in supplementary file (Figure S2).

The APTES contents of the LAP-APTES were calculated based on loss of weight between 150 and 600 °C minus the loss of weight of LAP in the same temperature range [49]. As expressed in Table S2, this content varies from 1.6% to 8.2% at 1 and 10 CEC of APTES, respectively. These percentages allow to calculate the amount of adsorbed APTES that can be both compared to the initial load of APTES and to the exchange capacity of edge-sites of LAP (i.e., 0.36 mmol g^−1^) [44]. Therefore, for the three highest loads of APTES, the adsorbed amount is higher to the number of edge-sites. An important drop of the grafting yield is also displayed after 3 CEC, from 57% to 30% at 3 and 10 CEC of APTES, respectively. This would imply a possible modification of the condensation between APTES and clay minerals for the highest APTES load, and/or the possible adsorption onto the surface of LAP due to the saturation of edge-sites.

The covalent binding of APTES with LAP can be evidenced by solid-state ^29^Si NMR spectroscopy. The different Si species are denoted according to T*^n^* and Q*^n^* notation where T and Q refer to tri- and tetra- monofunctional units respectively, and n is the number of bridging O–Si or O–Mg atoms surrounding the Si atom [50].

**Figure 3:**
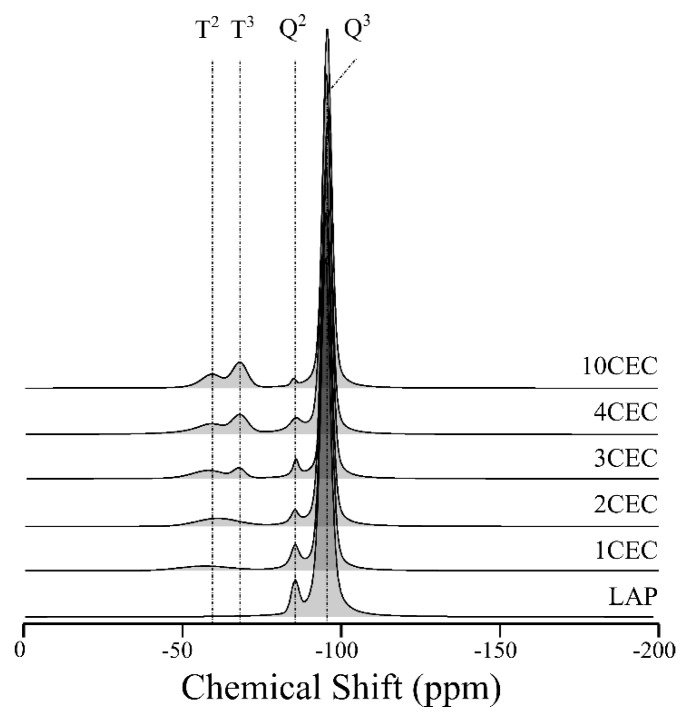
Solid-state ^29^Si NMR spectra of LAP and LAP-APTES for different loads of APTES.

Two resonances are displayed on the ^29^Si NMR spectrum of LAP at −95 and −85 ppm respectively (Figure 3). These resonances correspond to Q^3^ fully coordinated Si atoms and Q^2^ sites related to isolated silanol groups at the edge of clay minerals. The proper grafting of APTES can be testified by the relative decrease of the resonance Q^2^ intensity, indicating a decrease in isolated edge-sites [51]. In the same way, the appearance of T^2^ and T^3^ signals is an indicator of the grafting of APTES [52]. These two resonances are merged for the lowest loads of APTES (i.e., 1 and 2 CEC) and can be distinguished after 3 CEC (Figure 3).

Typically, T^3^-type environments indicate a high condensation of the silicon species from APTES (−66 ppm) whereas T^2^-type environments indicate the presence of less condensed species in the structure (i.e., silanol groups, (−57 ppm). Here, the T^3^/T^2^ ratio increases with the load of APTES indicating a better condensation (Table 1). The Q^3^/Q^2^ ratio also increases on increasing the load of APTES (Figure 3), indicating both the grafting of APTES and the decrease number of isolated Si in edge-sites. Therefore, the increase of the starting concentration of APTES seems to enhance the condensation of APTES onto LAP (i.e., relative), even if the amount of grafted surface sites gradually increases as well (Table 1).

**Figure 4:**
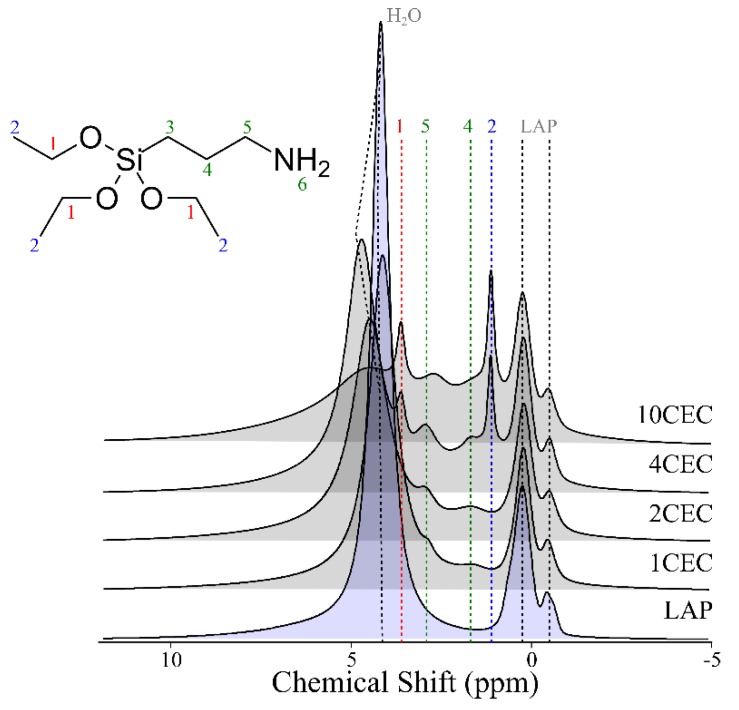
Solid-state ^1^H NMR spectra of LAP and LAP-APTES for different loads of APTES.

Solid-state ^1^H NMR spectra provide precious information on the degree of condensation of APTES. It allows for precise determination of the relative proportion of each type of ^1^H in the studied sample. As exhibited in Figure 4, LAP presents three different ^1^H signals. The first one is assigned to adsorbed water molecules (4.5 ppm) and the others are related to the structural –OH of LAP (i.e., −0.5 and 0.3 ppm) [53]. 

In the presence of APTES, two new resonances appear on the spectra. The first is attributed to the protons of the aminopropyl moieties (i.e., 4 and 5, Figure 4) and the second is related to triethoxy moieties (i.e., 1 and 2, Figure 4). From the former, a relative gradual increase from 1 to 10 CEC is observed, as well as for the latter. 

This confirms that the increase of the APTES load leads to an increase of the amount of adsorbed APTES. Yet, the condensation of APTES seems to be least performed as the load of APTES increases. This is not in agreement with the ^29^Si NMR results as the condensation tends to be better performed with higher APTES load. However, the strong increase of adsorbed APTES for these ratios can hinder the assessment of the condensation. Obviously, a strong modification of APTES conformation is performed between 2 and 4 CEC.

As previously observed on ^29^Si NMR spectra, there is an increase of the contribution of T type environments between 2 and 4 CEC, which leads to a strong increase of the resonance attributed to the protons from the aminopropyl moieties in ^1^H NMR spectra. Therefore, it should be considered that for the higher load of APTES, the condensation is well-performed on edge-sites, whereas other bindings (i.e., surface/interlayer) could occur resulting in this important increase in “1” and “2” resonance intensity (Figure 4).

The analysis of the ZP allows characterizing the global surface charge of the starting material and LAP-APTES. ZP of clay mineral is generally negative due to isomorphic substitution in tetrahedral and/or octahedral sheets [54]. However, the disk shape of LAP increases the relative number of edge-sites which could be protonated or not depending of the pH of the solution [55]. The pH_pzc_ of LAP edge-sites is 11 [56,57]. As a result, edge-sites are protonated in a wide range of pH, balancing the negative surface charge of LAP. Therefore, ZP values of LAP are close to zero for pH < 10, and become negative under strong alkaline pH conditions (Figure S3). Conversely, the ZP values of LAP-APTES are impacted by the speciation of APTES, in which –NH_2_ moieties are protonated (i.e., NH_3_^+^) under acidic pH conditions [58]. Therefore, the ZP of LAP-APTES is positive for pH values < 6. None impact of the load of grafting agents is displayed by the results. Under alkaline pH conditions, ZP values of LAP-APTES are negative in a similar extent whatever the load of APTES. Based on the results, the pH_pzc_ of LAP-APTES is assumed to be 6 (Figure S3), whereas the accurate estimation of pH_pzc_ of LAP is not possible.

### 3.2. Adsorption Experiments

#### 3.2.1. Impact of pH

**Figure 5:**
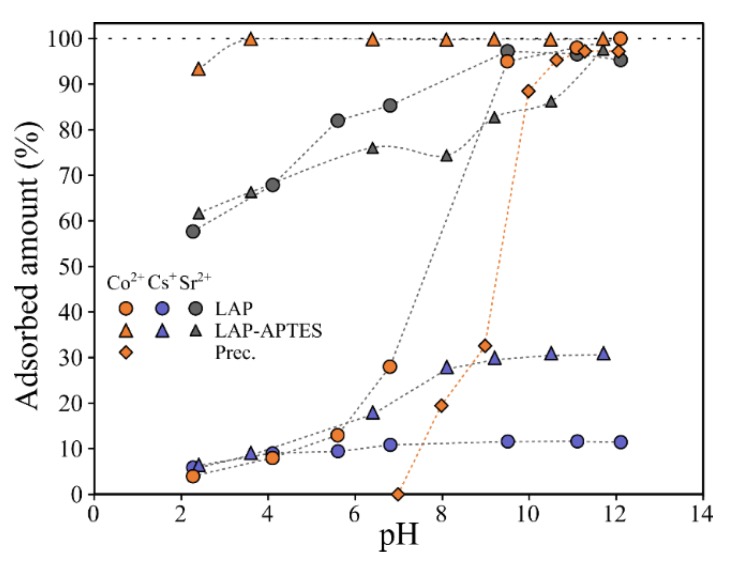
Impact of pH on the competitive adsorption of Cs^+^ (blue), Co^2+^ (orange) and Sr^2+^ (grey) onto LAP (squares) and LAP-APTES-4CEC (triangles). Diamonds represent the precipitated amount of Co^2+^.

The impact of the pH on the single-solute adsorption of Co^2+^, Cs^+^, and Sr^2+^ onto LAP and LAP-APTES-4CEC is presented Figure 5. In general, the increase of the pH is favorable for the adsorption of the targeted contaminants onto the adsorbents. As represented by the variation of the ZP values, the increase of the pH value results in a global negative surface charge of both LAP and grafted LAP occurs due to the deprotonation of edge-sites of LAP and the deprotonation of –NH_3_^+^ moieties of APTES [58]. This modification of the global charge of the adsorbent can diminish the repulsion between cationic contaminants and adsorbents, improving their adsorption [59].

This increase is especially noticeable on the adsorption of Sr^2+^ which is performed in a similar extent onto LAP and LAP-APTES. The adsorbed percentage ranges from 60% to 95% at pH = 2.1 and pH = 12.2 respectively (Figure 5). The adsorption of Cs^+^ is very limited onto LAP and the modification of the pH doesn’t affect in a significant extent its adsorption. Conversely, the adsorption of Cs^+^ onto LAP-APTES is enhanced under alkaline pH conditions (i.e., from 7% to 30%).

These results demonstrate the better affinity of Cs^+^ and Co^2+^ for LAP-APTES in comparison with LAP. As a result, the adsorption of the contaminants onto LAP will not be presented within this study while the impact of the APTES load onto this adsorption will be emphasized.

Co^2+^ is the contaminant that exhibits the most important adsorption variation between LAP and LAP-APTES. Its adsorption is limited onto LAP whatever the pH value (i.e., the high adsorbed amount onto LAP under alkaline pH conditions is overlapped by its precipitation), whereas its adsorption is almost complete in a wide range of pH onto LAP-APTES. The only pH value for which a significant equilibrium concentration is noticed at pH = 2.1, for which an important part of amines moieties is protonated.

#### 3.2.2. Single-Solute Adsorption Isotherms

The single-solute adsorption isotherms of Cs^+^ onto LAP-APTES are presented Figure S4. The adsorption capacities of each adsorbent range between 0.115 and 0.155 mmol g^−1^, without a clear trend with the variation of the load of APTES. The increase of the adsorbed amount is gradual and accompanied with a high increase of the equilibrium concentration. Moreover, the non-steady-state of the adsorbed amount can be noticed whatever the APTES load (Figure S4). The adsorption of Cs^+^ onto LAP-APTES is well-fitted by the Freundlich model which assumes that the uptake of adsorbate ions occurs on a heterogeneous adsorbent surface. The Freundlich model is empirical in nature which further assumes that the stronger binding sites are occupied first and that the binding strength decreases with increasing degree of site occupation. Therefore, it can be considered that the affinity between Cs^+^ and adsorbent is limited, as emphasized by the mean free energy determined with the DR equation with *E* < 8 kJ mol^−1^, indicating physisorption (Table 2).

**Figure 6:**
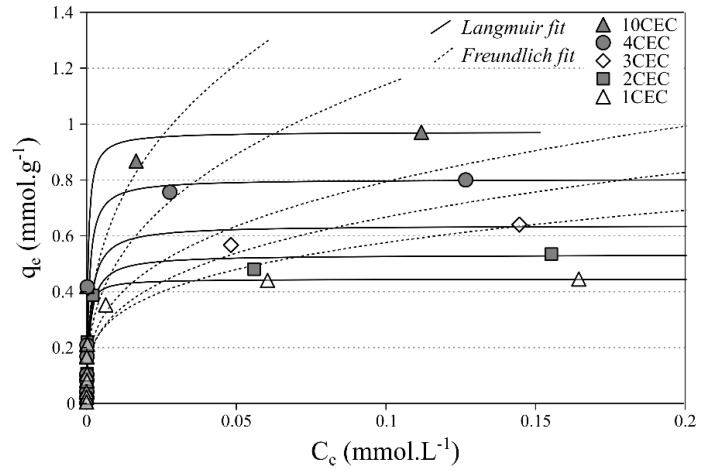
Single-solute adsorption isotherms at 293 K of Co^2+^ onto LAP-APTES for different loads of APTES (pH = 6–6.5).

The adsorption of Co^2+^ onto LAP-APTES exhibits a different pattern. Indeed, the adsorption isotherms show two distinct regimes with one displaying an important increase of adsorbed amount for the lower starting concentrations whereas the other exhibits a steady-state (Figure 6). The adsorption capacity, related to this steady-state of adsorbed amount, varied between each load of APTES. The lowest adsorption capacity is noticed at 1 CEC, with an adsorbed amount of 0.42 mmol g^−1^, whereas the highest adsorption capacities are noted at 10 CEC with an adsorbed amount of 0.98 mmol g^−1^. Therefore, the increase of the APTES load increases the adsorption capacity of Co^2+^.

The use of the adsorption models demonstrates a good agreement between experimental data and the Langmuir equation. As a reminder, this model assumes that adsorption occurs at specific homogeneous adsorption sites within the adsorbent. Therefore, the two distinct regimes exhibit, for the former, a strong affinity between Co^2+^ and the adsorbents and for the latter (i.e., steady-state) a saturation of the adsorption sites. From Langmuir and DR models, a good agreement between the modeled adsorption capacities and experimental ones is observed. Moreover, the mean free energies derived from DR equation are systematically higher than 8 kJ mol^−1^ indicating chemisorption (Table 2).

**Figure 7:**
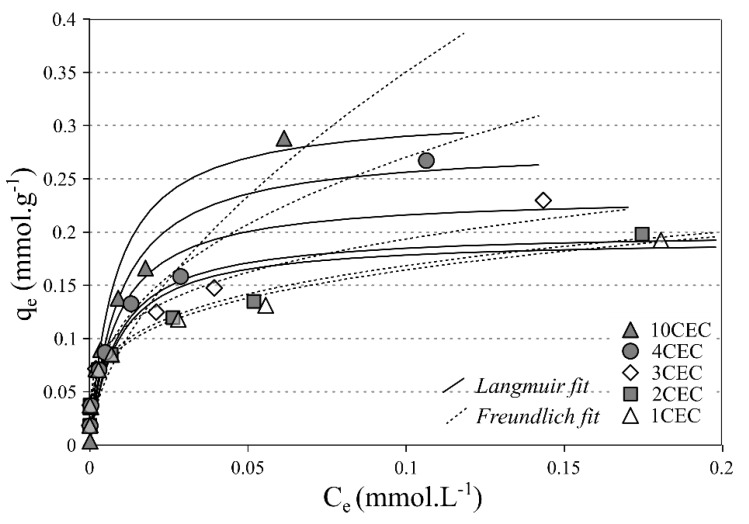
Single-solute adsorption isotherms at 293 K of Sr^2+^ onto LAP-APTES for different loads of APTES (pH = 6–6.5).

The single-solute adsorption isotherms of Sr^2+^ onto LAP-APTES are presented Figure 7. In the investigated range of concentrations, the non-steady-state is clearly reached, despite a good agreement with the Langmuir equation. Here, the increase of the adsorbed amount is regular and a strong increase of equilibrium concentrations is displayed for the two highest starting concentrations. The adsorption capacity is minimal at 1 CEC with 0.19 mmol g^−1^ and maximal at 10 CEC with 0.28 mmol g^−1^. Accordingly, the adsorption capacity of Sr^2+^ is higher for higher APTES load (Figure 7).

However, this variation in the adsorption capacities does not modify, to a significant extent, the affinity between the adsorbent and the adsorbate. Indeed, the experimental data are properly fitted by the tested models, emphasizing the hybrid characteristics of the adsorption of Sr^2+^ onto LAP-APTES in the used concentration range (i.e. none saturation reached). 

It should be highlighted that the *E* values from DR model are systematically higher than 8 kJ mol^−1^ and that adsorption can be considered as spontaneous (Table 2). Therefore, the adsorbed amount of Sr^2+^ increases for higher APTES load even adsorption properties with lower APTES load remain significant.

#### 3.2.3. Competitive Adsorption Isotherms

The competitive adsorption experiments of contaminants affect their adsorption in various extent (Figures S5–S7). In the tested concentration range, the adsorption of Co^2+^ is unaffected by the competition with Sr^2+^ and Cs^+^. The adsorption capacities remain equivalent and are almost complete whatever the load of APTES. Note that the fits with the models give absurd parameters due to the complete adsorption in the tested concentrations ranges. Therefore, any assumptions could not be done based on these results. However, we can compare the adsorption behavior of Co^2+^ in single-solute and in competition because of the same adsorption properties. It is only impossible to ensure any steady state and nor adsorption capacity during competitive experiments in the starting concentration range. 

Finally, the invisible effect of the competition with Cs^+^ and Sr^2+^ indicates specific adsorption sites for Co^2+^ or a better selectivity [12,60].

The adsorption capacity of Sr^2+^ is affected by the competition in the same extent whatever the load of APTES. A loss of 20% of adsorption capacity can be noticed, although the parameters derived from the model are comparable. The mean free energies remain higher than 8 kJ mol^−1^ and the other parameters do not vary to a significant extent. Therefore, it can be concluded that Sr^2+^ obviously competes with other contaminants for adsorption sites to a significant extent. However, the drop of the adsorption capacity remains moderate, indicating a good selectivity of the adsorbent for Sr^2+^.

Cs^+^ is at the same time the least adsorbed contaminant onto LAP-APTES and also the most impacted contaminant by the competition. The adsorption capacity in competition is indeed around a quarter of the adsorption capacity in single-solute solution (Table 2). Although in the latter, none steady state was reached, in the former, the adsorbed amount of Cs^+^ reach a plateau between 0.036 and 0.048 mmol g^−1^ depending on the adsorbent. One more time, no clear pattern can be displayed with the load of APTES. However, this steady state indicates a limiting factor for the adsorption of Cs^+^, which could be related to the adsorption of other contaminants, affected in a lower extent by the competition. As a result, the competitive adsorption isotherms of Cs^+^ are properly fitted by both Langmuir and Freundlich models whereas only Freundlich equation correctly fitted single-solute adsorption isotherms (Figure S3). However, the mean free energies derived from DR model remain inferior to 8 kJ mol^−1^, indicating physisorption as for single-solute experiments. This pronounced impact of the competition on the adsorption of Cs^+^ finally indicates an unfavorable adsorption behavior of Cs^+^ and a poor selectivity of the adsorbents.

### 3.3. Desorption Experiments

The desorption experiments were conducted in three saline solutions (i.e., pure water, 2 mM NaCl and 1 mM CaCl_2_) with LAP-APTES loaded with various amounts of APTES and the highest investigated starting concentrations of Co^2+^, Cs^+^, and Sr^2+^. 

**Figure 8:**
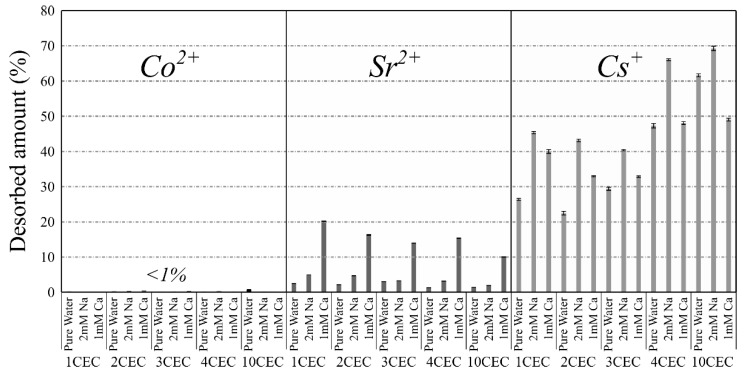
Desorption percentage of Co^2+^, Cs^+^, and Sr^2+^ as a function of the adsorbent and the releasing solution.

The results are presented in Figure 8. Desorption of Co^2+^ is not affected by the releasing solution or the load of APTES. Thus, desorption percentages after one week are systematically inferior to 1% with non-significant variations. Therefore, the adsorption of Co^2+^ is very stable in the tested conditions.

Conversely, the desorption percentage of Sr^2+^ increases from pure water to 1 mM CaCl_2_ with intermediate values in NaCl solution. Even if the desorption is low in pure water and NaCl solution (between 1% and 6%) with higher desorption in NaCl in comparison to pure water for a same load of APTES, the desorption percentage is significantly higher in CaCl_2_ solution (Figure 8). As a result, the valence of the inorganic cation in the releasing solution is of high concern about the desorption behavior of Sr^2+^. Generally, a decrease in the desorption percentage on increasing the load of APTES is also noticed. Therefore, the adsorption capacity is higher and desorption percentage lower for the highest load of APTES.

The pattern of Cs^+^ release is different. Cs^+^ is the only contaminant for which desorption is very significant even in pure water, with release percentages between 22% and 61 % (Figure 8). Systematically, the desorption percentage is the highest in the NaCl solution for a given load of APTES. It can be connected with the previous observation on the high desorption of Sr^2+^ in the presence of Ca^2+^ (Figure 8). Indeed, Na^+^ and Cs^+^ also have the same valence. Conversely to the desorption of Sr^2+^, the desorption of Cs^+^ increases with increasing the load of APTES. This indicates a variation in the binding energy with increasing the load of APTES, which would not be observed based on the adsorption isotherms.

### 3.4. Sorption Mechanisms

The adsorption of Sr^2+^ onto LAP-APTES is favorable with adsorption capacity that ranges from 0.19 to 0.29 mmol g^−1^ depending on the load of APTES. These values are systematically below the CEC of LAP (i.e., 75 meq 100 g^−1^), except for extreme pH values, at which the adsorption capacities are close. Moreover, desorption experiments have exhibited that the desorption percentage of Sr^2+^ was maximal in CaCl_2_ solution, i.e., an inorganic cation with the same valence. Conversely, the desorption rate was insignificant in pure water solution. These two patterns point out that Sr^2+^ is adsorbed through cation exchange onto LAP-APTES, following this reaction:

$$\equiv XNa+\;Sr^{2+}=XSr^{+}+Na^{+}$$

Yet, the increase of adsorption capacities with higher load of APTES raise question about the impact of grafted agents. It can be assumed that the better pillaring of clay sheets that is performed on increasing the load of APTES would be favorable for the availability of adsorption sites within the layers. However, the ligand adsorption of Sr^2+^ onto APTES can also be advanced to explain the higher adsorption capacities for higher APTES contents [38].

The adsorption of Cs^+^ onto LAP-APTES is very weak, independent to the load of APTES and very sensible to the competition with Co^2+^ and Sr^2+^. As a result, its adsorption is poorly selective and unfavorable onto the used adsorbents. Desorption experiments have also demonstrated that even if the desorption rate was maximal in NaCl solution, its release is also very important in pure water (from 20% to 60%). For higher load of APTES, the desorption percentage is higher for the same releasing solution. As a result, the adsorption of Cs^+^ onto LAP-APTES is probably carried out through cation exchange in a little extent, but most of Cs^+^ is adsorbed through weak electrostatic interactions on the external surface or edge-sites of LAP as described below.

$$\equiv XNa+\;Cs^{+}=XCs+Na^{+}\;\;\equiv SOH+Cs^{+}=SOCs+H^{+}$$

Therefore, the grafting of starting clay minerals did not enhance its adsorption in a significant extent.

Finally, the adsorption of Co^2+^ is probably the most interesting result of this work. The adsorption is indeed very favorable with insignificant equilibrium concentrations in a wide range of starting concentrations. The steady-state in adsorption is reached for different adsorbed amounts with a good correlation with the amount of adsorbed APTES, i.e., the higher the load of APTES, the higher the adsorption capacity of Co^2+^. The other interesting result on the binding between Co^2+^ and LAP-APTES is that its adsorption is not impacted by the competition with Cs^+^ and Sr^2+^, and its desorption is insignificant whatever the saline solutions. The adsorption of Co^2+^ onto LAP-APTES is therefore performed by a coordination bond with –NH_2_ moieties of APTES as expressed below.

$$\equiv RNH_{2}+Co^{2+}=RNH_{2}Co^{2+}$$

As a consequence, the silylated clay minerals display an excellent affinity for Co^2+^, which is higher than other adsorbents in the literature [12,16,59], whereas the adsorption capacity of Sr^2+^ are comparable with other adsorbents [9]. Conversely, LAP-APTES is not an appropriate adsorbent for Cs^+^, with both an unfavorable adsorption behavior and high desorption in the tested conditions.

## 4. Conclusions

In this study, silane grafting of LAP was successfully performed for different load of APTES. The increase of the grafted amount of APTES impacted the structural properties of the resulted adsorbent. Therefore, the degree of condensation as well as the binding mechanisms of APTES were affected by the increase of the adsorbed amount of APTES. Non-intercalation within the interlayer space of LAP was observed rather than a little penetration as exhibited by the weak increase of the basal spacings of LAP-APTES with increasing the load of APTES. The adsorption of Co^2+^ and Cs^+^ onto LAP-APTES was significantly higher than onto LAP, and the increase of the load of APTES positively impacts the adsorption capacities of Co^2+^ and Sr^2+^ (i.e., but the adsorption capacities of Sr^2+^ remain lower onto LAP-APTES than that onto LAP). The adsorption of Co^2+^ was very specific and capacitive for the higher loads of APTES, and performed though coordination bond. Moreover, the desorption in saline solutions can be considered as insignificant in the tested conditions. It is not the case for Sr^2+^ (mostly adsorbed through ion exchange) and Cs^+^, for which the presence of inorganic cation with the same valence strongly increased their release. As consequence, the synthetized material can be considered as a suitable adsorbent for the removal of Co^2+^ and Sr^2+^ from low-level radioactive wastes. Further tests need to be conducted on real effluents to assess the possible competition especially between Sr^2+^ and other cations. However, this study represents a step forward bringing an alternative solution for the decontamination of aqueous solutions contaminated by radionuclides.

## 5. Patents

The developed adsorbent and different preparation ways to clean the synthesis of the adsorbents and improve the recovery of radionuclides were patented. Patent n°FR3078268 (30/08/2019) and WO 2019/162638 (29/08/2019) [61].

## Figures and Tables

**Table 1:** Ratios obtained from the deconvolution of ^29^Si NMR spectra.

Adsorbent	Q^3^/Q^2^	T^3^/T^2^	ΣT	ΣQ/ΣT
LAP	10.2	-	0	-
1CEC	14.9	-	5.1	18.6
2CEC	20.9	-	7.2	12.8
3CEC	24.9	0.31	10.2	8.7
4CEC	29.4	0.48	14.1	5.3
10CEC	51.2	0.92	16.1	5.2

**Table 2:** Single-solute (SS) and competitive (Comp) adsorption model parameters derived from Langmuir, Freundlich, and DR equations for the adsorption of Co^2+^, Cs^+^ and Sr^2+^ onto LAP-APTES for different loads of APTES.

			Langmuir				Freundlich			Dubinin–Radushkevich		
	Load		Q_max_ mmol g^−1^	K_L_ L mmol^−1^	Δ*G°* kJ mol^−1^	r^2^	n	K_F_ L g^−1^	r^2^	Q_m_ mmol g^−1^	*E* kJ mol^−1^	R^2^
Cs^+^	1CEC	SS	0.168	5.8	−21.13	0.839	2.17	0.17	0.973	0.100	6.108	0.850
		Comp	0.046	27.9	−24.95	0.990	5.58	0.05	0.974	0.041	7.143	0.945
	2CEC	SS	0.165	4.9	−20.75	0.800	2.08	0.16	0.943	0.093	6.063	0.804
		Comp	0.048	13.6	−23.20	0.949	4.06	0.05	0.948	0.038	7.809	0.872
	3CEC	SS	0.214	3.0	−19.53	0.628	1.83	0.17	0.926	0.094	5.590	0.866
		Comp	0.041	13.6	−23.20	0.973	3.54	0.04	0.959	0.034	7.001	0.922
	4CEC	SS	0.184	6.0	−21.22	0.826	2.16	0.18	0.975	0.110	6.154	0.861
		Comp	0.043	20.8	−24.24	0.995	3.55	0.05	0.951	0.041	6.967	0.987
	10CEC	SS	0.156	3.9	−20.14	0.684	1.80	0.14	0.969	0.064	6.537	0.831
		Comp	0.054	12.1	−22.92	0.981	2.16	0.07	0.952	0.051	5.376	0.950
Sr^2+^	1CEC	SS	0.194	114.2	−28.38	0.977	3.96	0.29	0.981	0.162	12.127	0.969
		Comp	0.168	106.9	−28.22	0.978	4.13	0.25	0.974	0.145	11.952	0.973
	2CEC	SS	0.201	115.8	−28.42	0.977	4.01	0.30	0.988	0.162	12.500	0.967
		Comp	0.163	70.2	−27.20	0.974	3.83	0.23	0.998	0.127	11.625	0.942
	3CEC	SS	0.234	118.5	−28.47	0.970	3.93	0.35	0.994	0.175	12.700	0.952
		Comp	0.192	58.8	−26.77	0.976	2.48	0.39	0.990	0.179	8.333	0.981
	4CEC	SS	0.281	105.8	−28.20	0.966	2.61	0.65	0.986	0.255	9.449	0.974
		Comp	0.224	74.8	−27.35	0.977	2.61	0.47	0.994	0.206	8.980	0.985
	10CEC	SS	0.313	124.2	−28.59	0.957	1.71	1.35	0.868	0.336	8.771	0.990
		Comp	0.276	89.5	−27.79	0.963	2.41	0.68	0.997	0.252	8.909	0.975
Co^2+^	1CEC	SS	0.445	204.1	−29.80	0.999	3.78	0.58	0.856	0.447	10.314	0.968
		Comp	-	-	-	-	1.93	2.47	0.613	0.941	7.332	0.708
	2CEC	SS	0.532	84.2	−27.64	0.998	3.22	0.67	0.662	0.528	10.911	0.968
		Comp	-	-	-	-	1.57	5.57	0.662	1.375	6.836	0.746
	3CEC	SS	0.638	80.0	−27.52	0.998	3.09	0.79	0.626	0.647	10.426	0.958
		Comp	-	-	-	-	0.54	19,256.42	0.984	48.526	4.603	0.993
	4CEC	SS	0.803	124.5	−28.59	0.999	2.80	1.14	0.685	0.841	9.901	0.930
		Comp	-	-	-	-	0.54	21,504.12	0.986	50.164	4.593	0.994
	10CEC	SS	0.972	214.3	−29.92	0.999	2.87	1.55	0.774	1.011	10.314	0.933
		Comp	-	-	-	-	0.94	523.06	0.993	7.514	6.682	0.995

## Supplementary Materials

The following are available online at https://www.mdpi.com/1996-1944/13/3/572/s1, Table S1: General information about grafting agent, 3-aminopropyltriethoxysilane (APTES), Table S2: Quantitative data extracted from TG curves of LAP-APTES, with OM content the organic matter content, Figure S1: FTIR spectra of LAP and LAP-APTES synthetized with different loads of grafting agents for wavenumbers between 400 and 1600 cm^−1^ (B) and between 2500 and 3700 cm^−1^ (A), Figure S2: DTG curves of LAP and LAP-APTES for different loads of APTES, Figure S3: Zeta potential (ZP) of LAP, LAP-APTES-4CEC and LAP-APTES-10CEC as a function of pH, Figure S4: Single-solute adsorption isotherms of Cs^+^ onto LAP-APTES for different loads of APTES (pH = 6–6.5), Figure S5: Competitive adsorption isotherms of Co^2+^ onto LAP-APTES for different loads of APTES (pH = 6–6.5), Figure S6: Competitive adsorption isotherms of Sr^2+^ onto LAP-APTES for different loads of APTES (pH = 6–6.5) and Figure S7: Competitive adsorption isotherms of Cs^+^ onto LAP-APTES for different loads of APTES.
