# Supplementary material for: Laponites® for the Recovery of 133Cs, 59Co, and 88Sr from Aqueous Solutions and Subsequent Storage: Impact of Grafted Silane Loads
Source: Materials (Basel). 2020 Jan 25;13(3):572. doi: 10.3390/ma13030572 (PMC7040832; doi:10.3390/ma13030572)
Supplement: Supplementary file 1 [file materials-13-00572-s001.pdf]

materials

Supplementary

Laponites® for the Recovery of ^{133}Cs, ^{59}Co and ^{88}Sr from Aqueous Solutions and Subsequent Storage: Impact of Grafted Silane Loads

Thomas Thiebault [1,2,3,*], Jocelyne Brendlé [1,2], Grégoire Augé [4] and Lionel Limousy [1,2]

[1] IS2M, Université de Haute-Alsace, CNRS, UMR 7361, 3b rue Alfred Werner, F68100, Mulhouse, France; jocelyne.brendle@uha.fr (J.B.); lionel.limousy@uha.fr (L.L.)
[2] Université de Strasbourg, F-67081, Strasbourg, France
[3] EPHE, PSL University, UMR 7619 METIS (SU, CNRS, EPHE), 4 Place Jussieu, F-75005, Paris, France
[4] ONET Technologies, 36 Boulevard de l'Océan, CS 20280, 13258, Marseille Cedex 09, France; gauge@onet.fr
* Correspondence: thomas.thiebault@ephe.psl.eu; Tel.: +33-(0)-144-27-59-97

Received: 20 December 2019; Accepted: 22 January 2020; Published: 25 January 2020

Table S1. General information about grafting agent, 3-aminopropyltriethoxysilane (APTES).

**Structure**	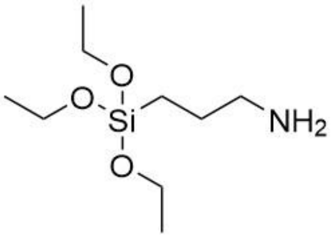
Formula	$C_9H_{23}NO_3Si$
CAS-Number	919-30-2
Mass weight	221.37 g mol^{-1}

Table S2. Quantitative data extracted from TG curves of LAP-APTES, with OM content the organic matter content.

**Load of APTES**	**OM Content [%]**	**APTES Content [mmol g^{-1}]**	**Grafting Yield [%]**	**Edge-Sites Occupation [CE_{edges}]**
1 CEC	1.6	0.14	59.2	0.38
2 CEC	3.2	0.26	56.8	0.73
3 CEC	4.7	0.39	56.6	1.09
4 CEC	5.5	0.47	50.6	1.30
10 CEC	8.2	0.68	29.6	1.90

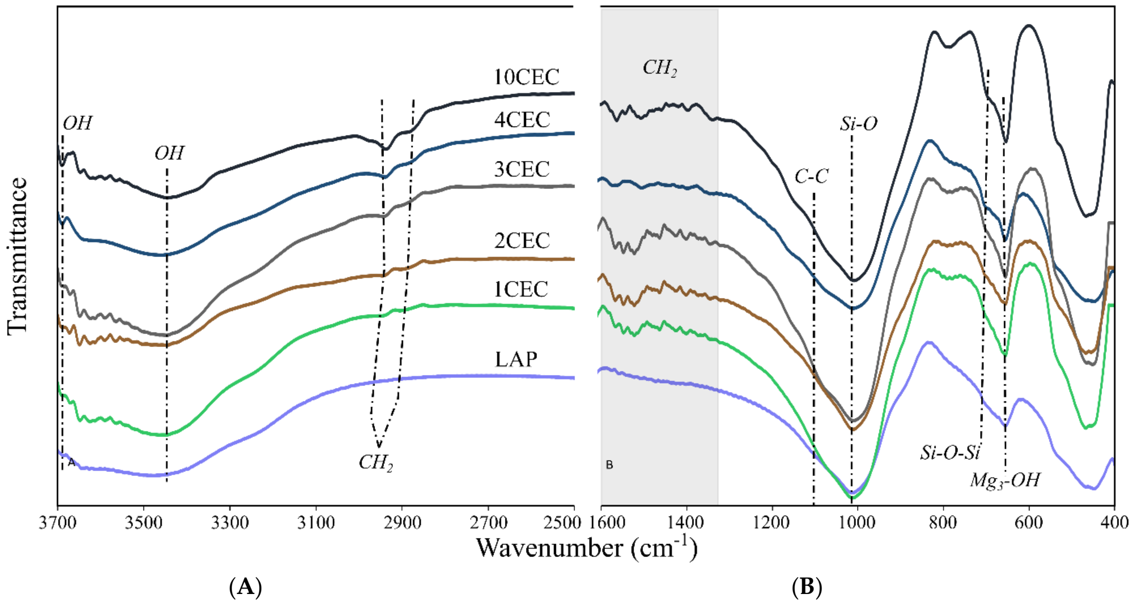

Figure S1. FTIR spectra of LAP and LAP-APTES synthetized with different loads of grafting agents for wavenumbers between 400 and 1600 cm^{-1} (**B**) and between 2500 and 3700 cm^{-1} (**A**).

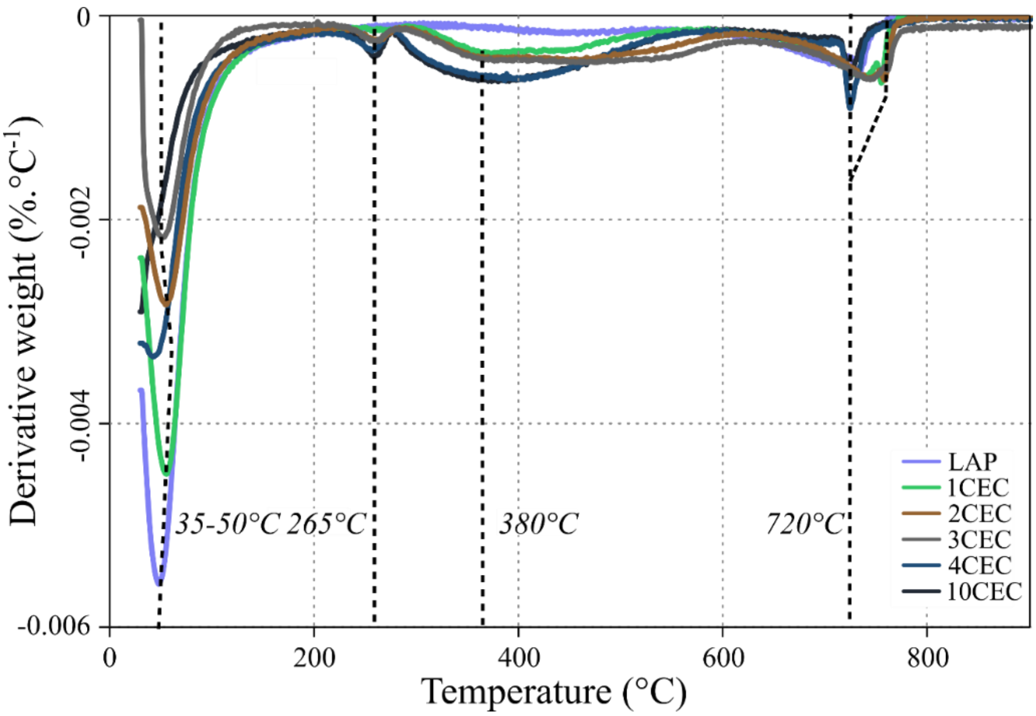

Figure S2. DTG curves of LAP and LAP-APTES for different loads of APTES.

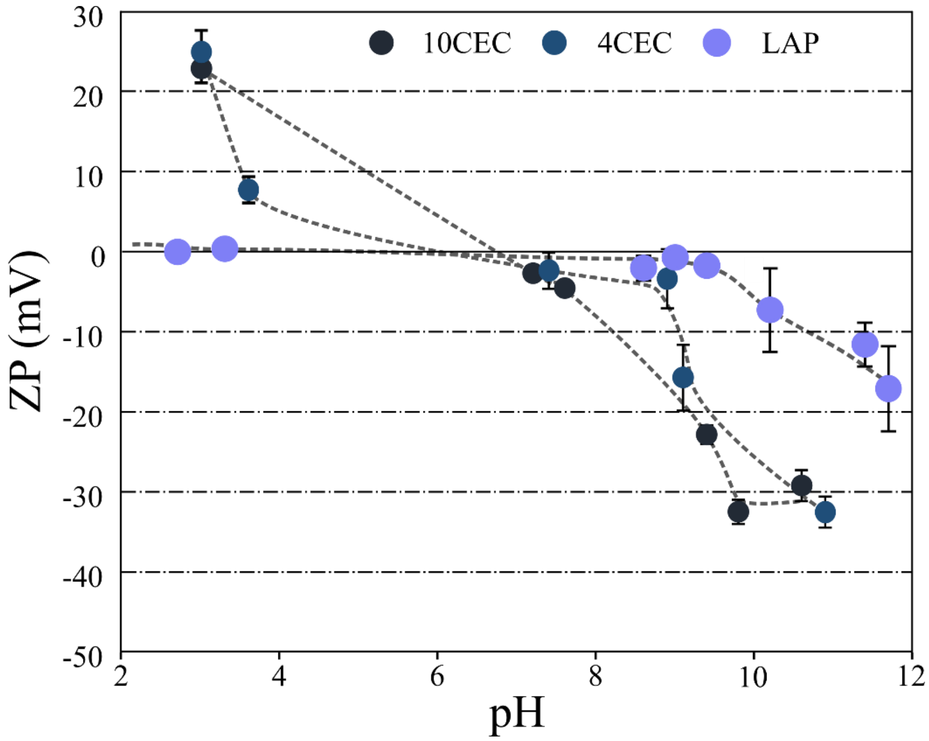

Figure S3. Zeta potential (ZP) of LAP, LAP-APTES-4CEC and LAP-APTES-10CEC as a function of pH.

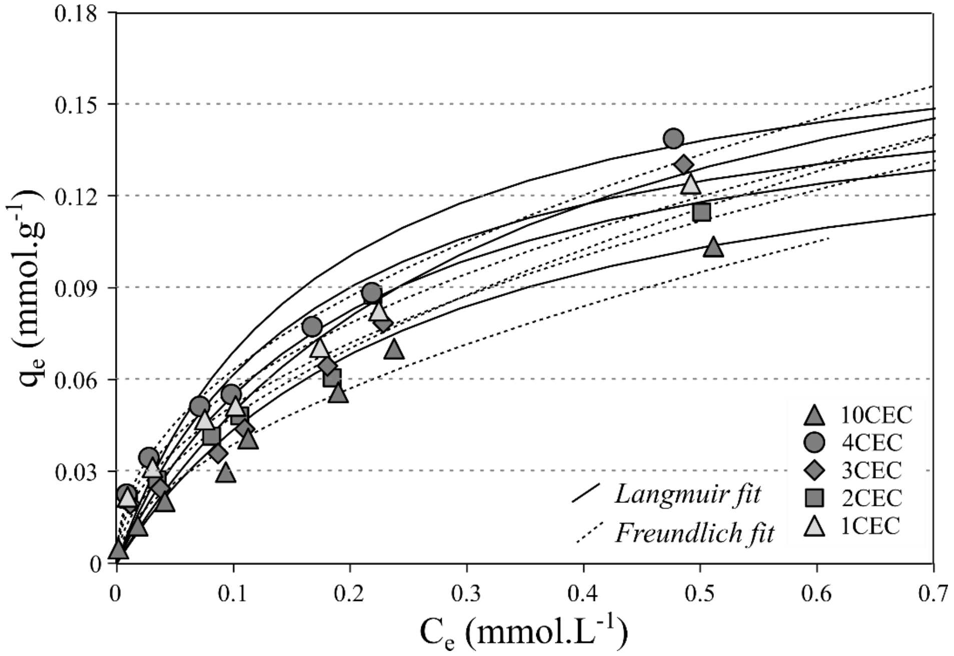

Figure S4. Single-solute adsorption isotherms at 293 K of Cs^+ onto LAP-APTES for different loads of APTES (pH = 6–6.5).

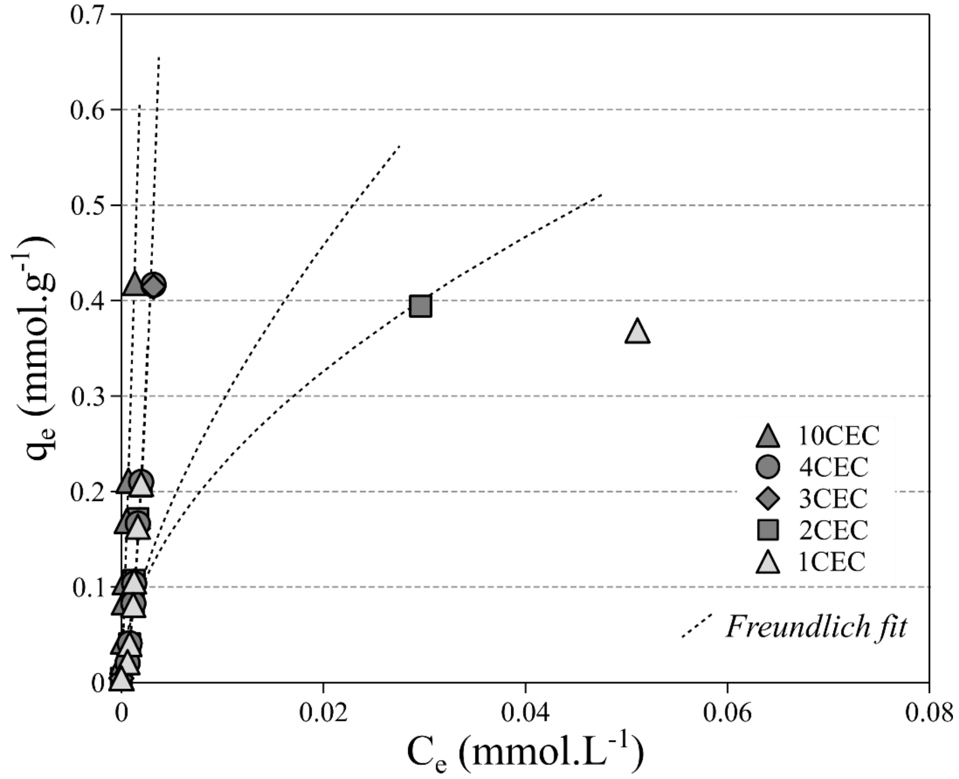

Figure S5. Competitive adsorption isotherms at 293 K of Co^{2+} onto LAP-APTES for different loads of APTES (pH = 6–6.5).

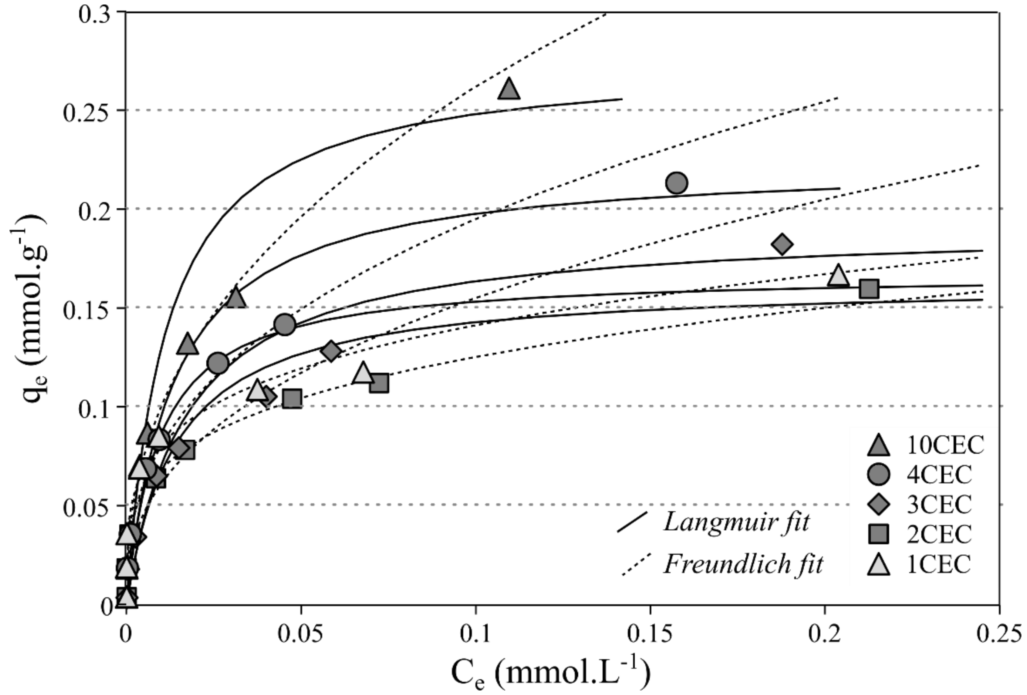

Figure S6. Competitive adsorption isotherms at 293 K of Sr^{2+} onto LAP-APTES for different loads of APTES (pH = 6–6.5).

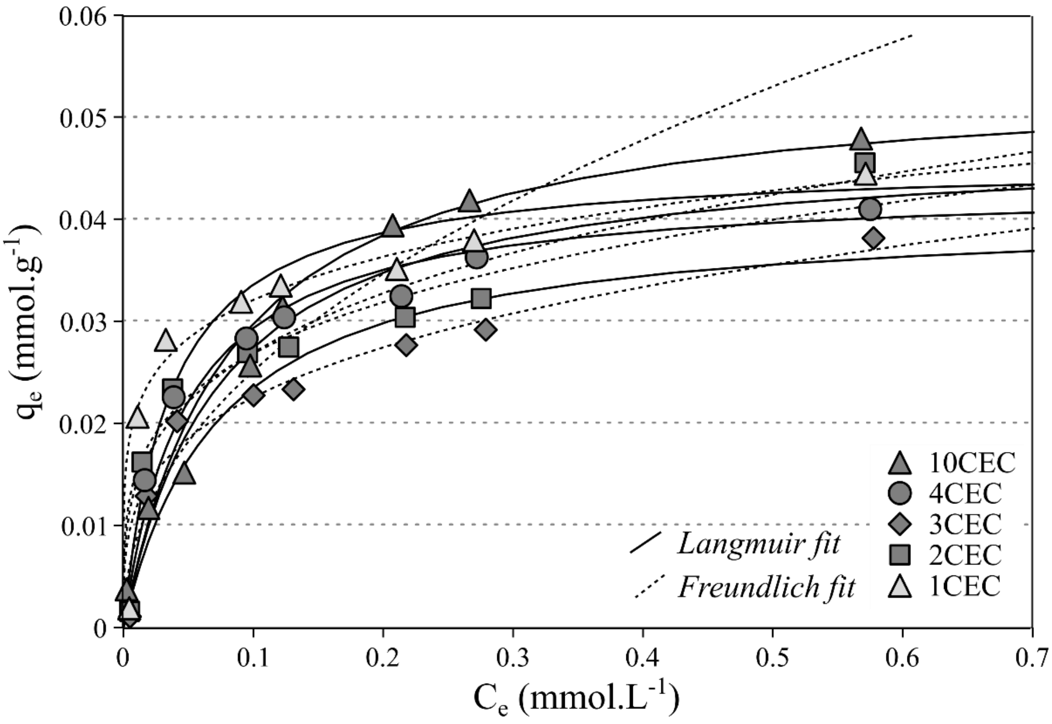

Figure S7. Competitive adsorption isotherms at 293 K of Cs^+ onto LAP-APTES for different loads of APTES (pH = 6–6.5).

© 2020 by the authors. Licensee MDPI, Basel, Switzerland. This article is an open access article distributed under the terms and conditions of the Creative Commons Attribution (CC BY) license (http://creativecommons.org/licenses/by/4.0/).
